# Electro-mechano responsive elastomers with self-tunable conductivity and stiffness

**DOI:** 10.1126/sciadv.adf1141

**Published:** 2023-01-25

**Authors:** Guolin Yun, Tim Cole, Yuxin Zhang, Jiahao Zheng, Shuaishuai Sun, Yiming Ou-yang, Jian Shu, Hongda Lu, Qingtian Zhang, Yongjing Wang, Duc Pham, Tawfique Hasan, Weihua Li, Shiwu Zhang, Shi-Yang Tang

**Affiliations:** ^1^ CAS Key Laboratory of Mechanical Behavior and Design of Materials, Department of Precision Machinery and Precision Instrumentation, University of Science and Technology of China, Hefei, China.; ^2^ Cambridge Graphene Centre, University of Cambridge, Cambridge, UK.; ^3^ Department of Electronic, Electrical, and Systems Engineering, University of Birmingham, Birmingham, UK.; ^4^ School of Mechanical, Materials, Mechatronic, and Biomedical Engineering, University of Wollongong, Wollongong, Australia.; ^5^ Department of Mechanical Engineering, University of Birmingham, Birmingham, UK.

## Abstract

Materials with programmable conductivity and stiffness offer new design opportunities for next-generation engineered systems in soft robotics and electronic devices. However, existing approaches fail to harness variable electrical and mechanical properties synergistically and lack the ability to self-respond to environmental changes. We report an electro-mechano responsive Field’s metal hybrid elastomer exhibiting variable and tunable conductivity, strain sensitivity, and stiffness. By synergistically harnessing these properties, we demonstrate two applications with over an order of magnitude performance improvement compared to state-of-the-art, including a self-triggered multiaxis compliance compensator for robotic manipulators, and a resettable, highly compact, and fast current-limiting fuse with an adjustable fusing current. We envisage that the extraordinary electromechanical properties of our hybrid elastomer will bring substantial advancements in resilient robotic systems, intelligent instruments, and flexible electronics.

## Teaser

Conductive elastomers filled with solid-liquid transformable metal particles achieve self-adjustable resistance and stiffness.

## INTRODUCTION

Electrical conductivity and mechanical stiffness are two fundamental properties of any material system. These properties are usually not tunable, with materials and overall design chosen to satisfy the functional criteria defined by specific applications. However, an increasing number of emerging applications in robotics, structural engineering, and consumer wearable electronics would benefit from materials whose properties can be actively tuned (*1*–*6*). These smart materials that can respond to changes in their environment are revolutionizing products and devices. For instance, materials with switchable conductivity show promising applications in electronic switches (*7*), optoelectronics (*8*), random access memory (*9*, *10*), and reconfigurable electronics (*11*). In addition, materials with variable stiffness offer exciting opportunities in soft robotics and manipulators (*12*, *13*), automotive and aerospace engineering (*14*, *15*), and surgical and rehabilitation devices (*16*, *17*).

Metal-polymer conductive elastomers offer such tunable electrical or mechanical properties (*18*–*20*). Among them, conductive elastomers filled with low–melting point alloys [LMPAs; such as eutectic gallium (Ga)–indium and Field’s metal (FM, melting point: 62°C): eutectic tin–bismuth–indium] have recently become attractive because their resistance and stiffness can be changed by varying the temperature (*21*–*23*). These LMPAs have high electrical/thermal conductivity and high deformability after melting (*24*). The solidification of LMPAs can induce volume expansion, thereby contacting adjacent particles to transform an insulating composite into a conductor (*22*). Alternatively, melted LMPAs can fill the microgaps in conductive networks as the temperature rises, making the composite conductive (*21*). In addition, the melting of LMPA particles can reduce the stiffness of the composites to up to two orders of magnitude (*25*, *26*).

However, LMPA-filled elastomers demonstrated to date suffer from significant limitations (*27*). On one hand, composites filled only with LMPA fillers can only switch between conductive and insulating states. Therefore, they are not competent for more complex applications requiring continuous resistance changes, such as sensors. On the other hand, LMPA-filled elastomers usually require external control systems to change their temperature, such as liquid nitrogen immersion (*22*) or adjusting the applied electrical current (*23*, *25*). We note that existing works have not attempted to combine variable resistance and stiffness properties and investigate their synergistic effects to achieve independence from external control.

**Fig. 1.**
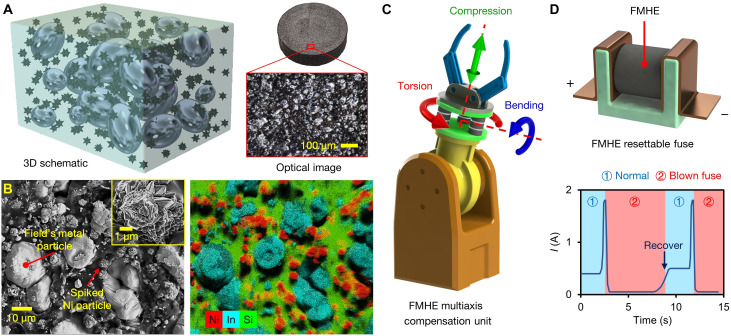
Microstructure of the FMHE and two applications based on it. (**A**) 3D schematic of the composition and optical microscope image showing the surface structure of the FMHE. (**B**) SEM and EDS images of the cross section of the FMHE_3_ sample. The distribution of Ni and FM particles; PDMS are represented by their characteristic elements of Ni, In, and Si. The inset shows a close-up SEM image of a spiked Ni particle. (**C**) An FMHE variable stiffness compensation unit (highlighted in green) mounted on a robot manipulator. (**D**) Schematic diagram of the FMHE resettable current-liming fuse and its current-time curve during cyclic operation.

Here, we create a stretchable FM-filled hybrid elastomer (FMHE) comprising hybrid fillers of FM and spiked nickel (Ni) microparticles dispersed in a polydimethylsiloxane (PDMS) matrix (Fig. 1, A and B). The use of FM avoids the extremely low solidifying temperature (below −30°C, caused by supercooling) required for Ga-based LMPAs (*28*, *29*). It is also biocompatible as it contains only indium, bismuth, and tin without lead or cadmium (*30*). The use of spiked metal particles as the secondary filler gives high strain sensitivity and improves microstructural strength and mechanical stability (*31*, *32*). The FMHE exhibits not only reversibly switchable stiffness but also tunable electrical properties. Harnessing these effects synergistically, we demonstrate a self-triggered variable stiffness compliance compensator for robotic manipulators, with 10 times improved compensation capabilities than the best commercial products (Fig. 1C). We also design a highly compact (>one order of magnitude smaller) self-recovering resettable current-limiting fuse with >10 times faster response than state-of-the-art resettable fuses (Fig. 1D).

## RESULTS

### Preparation of the FMHE

Figure 1A shows a three-dimensional (3D) schematic and the surface structure of a fabricated FMHE sample. A detailed preparation process is given in Materials and Methods and fig. S1. To prepare the FMHE, we first mix Ga-doped FM alloy [Ga content of 1 weight percent (wt%)] with PDMS in an 80°C water bath with a volume ratio of 1:1. The addition of Ga can form a Ga oxide layer on the surface of the FM droplet as it can provide the lowest Gibbs free energy (see fig. S2) (*33*, *34*). This prevents the coalescence of FM microdroplets during mixing and provides better control over the droplet size. The obtained FM-PDMS dispersion is then mixed with Ni microparticles and thermally cured in molds to obtain dark gray FMHE samples (Fig. 1A, right inset).

We prepare a batch of FMHE samples with different Ni/PDMS mass ratios of 0/0.5/1/2/3:1, denoted as FMHE_0/0.5/1/2/3_. Figure 1B shows scanning electron microscope (SEM) and energy-dispersive x-ray spectroscope (EDS) images of the cross section of the FMHE_3_ sample. The diameter of the FM particles in the FMHE_3_ is 10 to 20 μm. The spiked Ni microparticles increase the total volume of the FMHE composite and thus reduce the volume fraction of FM particles from 50 to 42.8%. They also separate FM particles to prevent them from contacting, as indicated by the thick PDMS layer between adjacent FM particles. We confirm this by observing the FM particles in FMHE_0_, which shows that the particles are in close contact with nanometer-wide gaps in between because of the absence of the Ni microparticles; see the SEM and EDS images in fig. S2. Because of the presence of these microparticles, the FMHE_3_ is expected to have a higher electrical resistivity at zero strain than that of the FMHE_0_. The inset in Fig. 1B shows the microstructure of an individual Ni particle with numerous spikes on the surface. The low–melting point FM and spiked Ni particles impart variable electromechanical properties and unconventional electrical strain response to FMHEs.

### Electrical properties

**Fig. 2.**
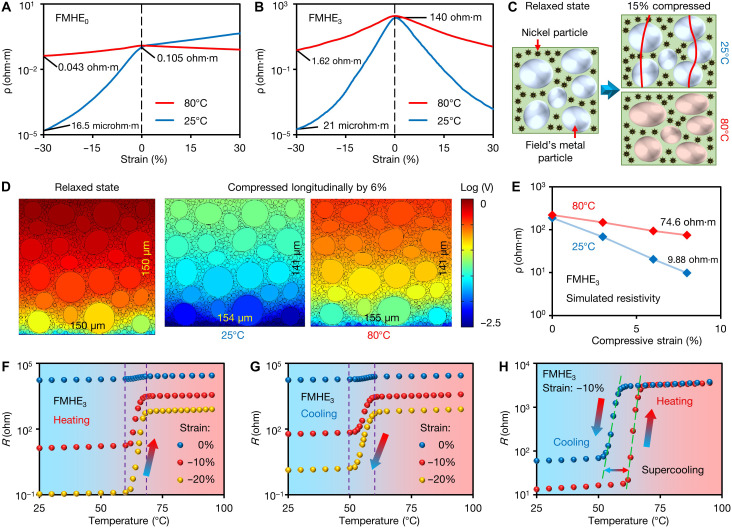
Electrical properties of the FMHE. Resistivity-strain curves of (**A**) FMHE_0_ and (**B**) FMHE_3_ at 25° and 80°C. A negative value of strain represents compression. (**C**) Illustrative schematics comparing the change in the filler network of the FMHE_3_ compressed by 15% at 25° and 80°C. (**D**) Simulation results of the electrical potential distribution of the FMHE_3_ at different temperatures. A larger potential drop (red color) corresponds to higher resistance. (**E**) Numerical simulated resistivity-strain curves of the FMHE_3_ at 25° and 80°C. Resistance-temperature curves of the FMHE_3_ sample under different strains during (**F**) heating, (**G**) cooling, and (**H**) a heating-cooling cycle under −10% strain.

We first investigate the electrical properties of the FMHE under mechanical deformation at different temperatures. Figure 2 (A and B) shows the resistivity-strain curves of FMHE_0_ and FMHE_3_ at 25°C (FM is solid) and 80°C (FM is liquid), respectively. The FMHE_0_ has an initial resistivity of 0.105 ohm·m at 25°C. Under compression, the FM particles are brought into contact, leading to an exponentially enhanced conductivity. For FMHE_3_, the Ni particles serving as spacers increase the initial resistivity to 140 ohm·m. The reduction in its resistivity under compression is more obvious than that of FMHE_0_, indicating an improved strain sensitivity. We suggest that during stretching, the FMHE_0_ resistivity increases because of the separation of the FM particles (positive piezoresistivity). Conversely, Ni particles reverse the piezoresistivity of FMHE_3_ to negative when stretched. We have previously shown that this effect is due to the hybrid conductive particle network and the spiked shape of Ni particles (*35*, *36*). Note that although the elastomer studied in this work exhibits the negative piezoresistive effect we previously reported, the presented electro-mechano responsive property and the self-triggered tunability have not been found before, as elaborated in the following sections. The resistivity of the composite mainly depends on the number of electrical connection pathways in the conductive filler network. Because of its positive Poisson’s ratio, FMHE_3_ is always compressed in a certain direction under any mechanical load, including stretching. The FM and Ni particles in FMHE_3_ then squeeze each other along the compression direction, leading to a sharp increase in the number of electrical connections, thereby reducing the resistivity (*35*). Furthermore, the protruding spikes of Ni particles allow the fillers to be in contact with a higher probability when FMHE_3_ is deformed (*31*). As a result, the conductivity of FMHE_3_ increases during deformation, showing a much higher strain sensitivity compared with FMHE_0_. This is also reflected in the higher gauge factor of the FMHE_3_, as given in fig. S3, which quantifies the strain sensitivity. As a comparison, we prepare composites filled with spherical Ni particles of the same size. However, because of the much higher resistance between spherical particles than that between spiked particles, these composites are insulating in the relaxed state (resistivity at >10^6^ ohm·m).

At 80°C, the FM particles melt, increasing the initial resistivity and greatly reducing the strain sensitivity of the FMHE. The melted FM microdroplets deform along with the PDMS matrix under strain, avoiding their close contact under compression and separation under stretching. The optical microscope images in fig. S4 and movies S1 and S2 clearly demonstrate the space changes between FM particles during compression and stretching, as well as their deformation upon melting. However, FMHE_3_ still shows better strain sensitivity as solid Ni particles can squeeze PDMS layers and form conductive paths. Figure 2C depicts the filler network changes in FMHE_3_ during compression at different temperatures. At 25°C, the thickness of the PDMS layers along the conductive pathways (red curves in Fig. 2C) decreases sharply when compressed, leading to a rapid decrease in resistivity. At 80°C, FM melts into droplets, while solid Ni particles can still squeeze PDMS layers and form conductive paths. Therefore, the resistivity of FMHE_3_ gradually decreases during compression, showing a lower strain sensitivity than that at 25°C.

To further verify our hypothesis, we perform finite element simulations of the resistivity of the FMHE_3_ compressed at different temperatures (see fig. S5 for detailed simulation settings). The simulation calculates the resistivity based on the drop of electrical potential by applying a fixed current density. The 2D model can qualitatively reveal the microstructural changes and predicts the sensitivity of FMHE at different temperatures (Fig. 2D). The deformation of FM droplets at 80°C is evident. The simulated resistivity-strain curves given in Fig. 2E show higher initial resistivity and lower sensitivity of FMHE_3_ at 80°C, which is consistent with the experimental results. The simulations on FMHE_0_ are also in line with the experimental conclusions (fig. S6).

We then investigate the influence of Ni and FM on the electrical properties of FMHEs; we find that their resistivity and strain sensitivity increase rapidly with the Ni content (fig. S7). Their resistivity also shows an obvious negative correlation with the particle size and volume fraction of FM (fig. S8), which is due to the increased opportunity to form additional conductive pathways when increasing the size of particle and volume fraction. Moreover, FMHE_3_ demonstrates good stability of the electrical property under cyclic loading tests at different temperatures; see fig. S9.

We also investigate the resistance-temperature relation of the FMHE_3_ samples (8 mm by 8 mm by 8 mm) during heating and cooling in Fig. 2 (F and G), respectively. Mainly because of the thermal expansion of PDMS (expansion rate of 0.034% °C^−1^) (*37*), the resistance of FMHE_3_ samples increases by 20 to 30% when the temperature rises from 25° to 60°C (Fig. 2F; see detailed discussion in text S2). As the samples are further heated, the FM particles gradually melt and deform under pressure, leading to their separation from surrounding conductive particles to induce a sharp increase in resistivity. The resistance stabilizes when all FM particles are melted. Our experiments show that this resistance change mainly depends on the temperature and is not significantly related to the heating rate or external mechanical manipulation. The resistance curve of the FMHE_3_ during cooling shows a decrease in resistance at a lower temperature compared to that shown during heating. This is because of a lower solidifying temperature of FM due to supercooling (*28*, *38*). From the differential scanning calorimetry curve of FM in fig. S10, we observe that the freezing point of FM is ~8°C lower than its melting point. Under compression, the resistance of the FMHE_3_ cannot return to its initial value after the FM resolidifies, as shown by the resistance-temperature curves in a heating-cooling cycle under 10% compressive strain (Fig. 2H). Because the FMHE_3_ sample is compressed, the FM droplets will solidify in a deformed shape during cooling. This increases the distance between conductive particles, leading to a higher initial resistance at 25°C. As a comparison, fig. S11 gives the resistance-temperature curves for FMHE_0_, which shows a similar phenomenon of the increase in resistivity when FM melts. In summary, both the resistivity and strain sensitivity of the FMHE can be tuned by temperature.

### Mechanical and electromechanical properties

**Fig. 3.**
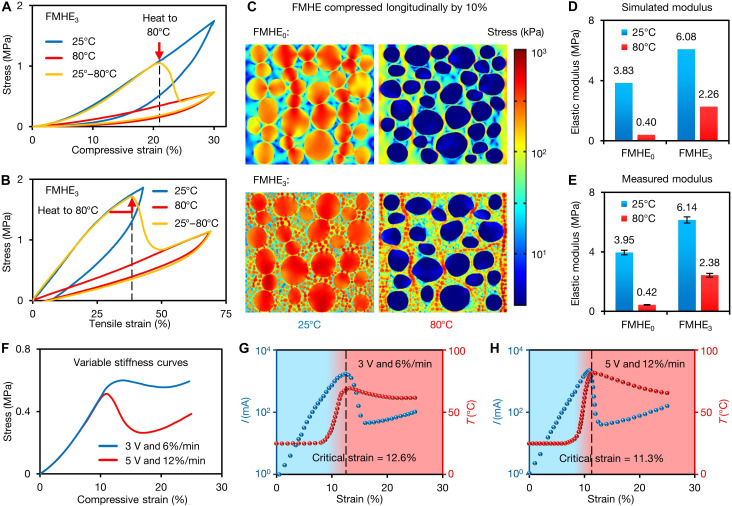
Mechanical properties of FMHEs. Stress-strain curves of the FMHE_3_ at different temperatures during (**A**) compression and (**B**) stretching. (**C**) Simulation results of the stress distribution of the FMHE_0_ and FMHE_3_ compressed by 10% at 25° and 80°C. (**D**) Simulated and (**E**) measured compressive modulus of FMHE_0_ and FMHE_3_ at 25° and 80°C. The values of the error bars are the SD of the modulus under five measurements. (**F**) Variable stiffness stress-strain curves of the FMHE_3_ powered by a fixed voltage during compression. Current-strain and temperature-strain curves of the FMHE_3_ powered by a fixed voltage during compression with different voltages and compression speeds of (**G**) 3 V and 6% min^−1^ and (**H**) 5 V and 12% min^−1^. The blue and red shaded regions correspond to the solidification and melting state of FM, respectively.

In addition to the temperature-dependent electrical properties, the FMHE samples also exhibit variable mechanical properties. Figure 3 (A and B) presents the stress-strain curves for the FMHE_3_ at different temperatures, showing stress softening of ~67% when FM melts. Each test involves heating the sample to 80°C while it is under stress. After the FM particles melt, the stress curve immediately moves to overlap the stress-strain curve at 80°C. The stress-strain curves demonstrate that FMHE_3_ exhibits elastic hysteresis behavior in both tension and compression. However, the FMHE_3_ shows significantly reduced elastic hysteresis at 80°C due to lower frictional energy losses (see details in fig. S12) (*39*). The consistent cyclic stress-strain curves shown in fig. S12 obtained from cyclic compression/stretching experiments reflect a high degree of mechanical stability of FMHE_3_ at both low and high temperatures. In addition, the stress-strain curves of FMHE_0_ (fig. S13) indicate its similar variable stiffness effect; however, it can be an order of magnitude softer at 80°C.

We also conduct numerical simulations to explain the stiffness softening of FMHEs at a high temperature (Fig. 3C). Under compression at 25°C, the stress is primarily distributed in solid FM particles (red color). At 80°C, FM melts into droplets with the internal stress approaching zero (blue color)—this explains the corresponding stress reduction. According to the simulated stress distribution and measured stress-strain curves, we calculate the compressive modulus of FMHE_0/3_ at 25° and 80°C (Fig. 3, D and E). The simulation results are consistent with the experiments, showing an error of less than 5%. After FM melts, the modulus of the FMHE_0_ and FMHE_3_ decrease by 89 and 62%, respectively, indicating a more pronounced stiffness reduction in FMHE_0_. The stress-strain curves for Ni-PDMS elastomers without FM at 25° and 80°C are only marginally different (fig. S14), indicating that the change in the FMHE stiffness is attributed to the phase transition of FM. Our experiments further show that the variable stiffness effect of FMHE weakens with increasing Ni content (fig. S15) but enhances with increasing FM particle size and content (fig. S16).

The unusual electromechanical properties of the FMHE can be harnessed to achieve a self-responsive stiffness tuning effect. We apply a dc voltage to an FMHE_3_ sample (8 mm by 10 mm by 10 mm) during compression to investigate the stiffness tunability (see fig. S17 for the experimental setup). Figure 3F gives the measured stress-strain curves under two conditions: low voltage–slow compression speed and high voltage–fast compression speed. Figure 3 (G and H) shows changes of current through the sample and its temperature for these two cases, respectively. At low strain, the current passing through the sample is low because of its high resistance. With an increase in strain, the resistance decreases until the current is high enough to melt the FM. This then causes a sharp decrease in stiffness and a rapid fall of stress. Figure 2F shows that melting the FM causes the resistance to increase, reducing the current and preventing a further rise in temperature. Under further compression, the current increases slowly again to maintain the melting of FM. According to Fig. 3F, the timing and speed of stiffness reduction of FMHE samples can be controlled by voltage and compression speed applied to them (see details in text S2 and fig. S18), realizing the electro-mechano responsive self-triggered variable stiffness function.

### Applications of the FMHE

**Fig. 4.**
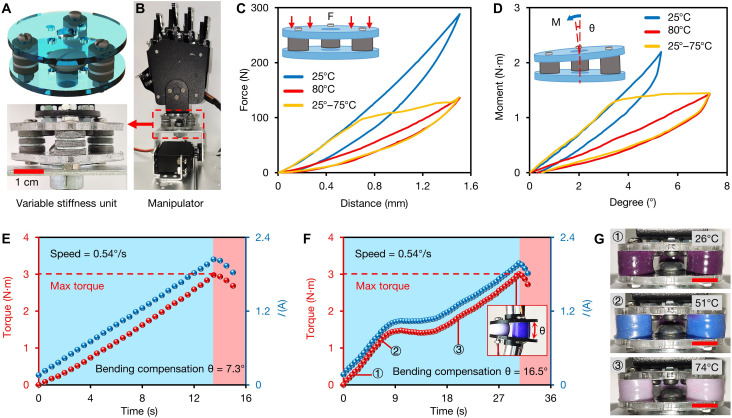
Self-sensing variable stiffness compliance compensator based on the FMHE_3_. (**A**) Schematic diagram and optical image of the FMHE compensation unit. (**B**) Photograph of the servomotor-driven manipulator equipped with a compensator. (**C**) Force-distance curves of the compensation unit at different temperatures during compression. (**D**) Moment-degree curves of the compensation unit at different temperatures during bending. (**E** and **F**) Torque-time and current-time curves of the digital servomotor that drives a robotic manipulator equipped with the compensation unit when the manipulator is stuck; the unit is powered by (E) 0-V and (F) 5-V voltage. The blue and red shaded regions correspond to the normal operation and blocked-rotor state (the servomotor is blocked due to excessive torque), respectively. The inset shows the photo of the bent compensation unit. (**G**) Photographs of the thermochromic elastomer-encapsulated FMHE compensator at different temperatures. Scale bars, 1 cm.

The unconventional electromechanical properties of the FMHE can solve many practical engineering challenges. To demonstrate its capabilities, we first harness its self-triggered variable stiffness property to develop a compliance compensation unit. Such a device could help a robotic manipulator to compensate for positional errors through its deformation, thereby preventing damage to tools or workpieces in tasks involving uncertain operational environments and complex contact scenarios. This is the key to robotic manipulation requiring a high-level dexterity where motions and positions must be compensated on multiple axes (*40*, *41*). However, state-of-the-art devices cannot provide adjustable stiffness and therefore have limited compensation ability. To address this deficiency, we design a FMHE variable stiffness compensator that can realize compression, bending, and torsion with variable and adjustable stiffness. Figure 4 (A and B) illustrates the structure of this device and a robotic manipulator equipped with it (see fig. S19 for the detailed structure). Figure 4 (C and D) presents its variable stiffness mechanical load curves during compression and bending, showing its different compressive/bending stiffness coefficients at 25° and 80°C. When powered by a 5-V dc supply, its stiffness automatically decreases under critical deformation because of the melting of FM. For the case of bending, its bending stiffness coefficient drops sharply from 0.413 N·m/° to almost zero at a bending angle of 3.7° (see details in text S3).

In our demonstration, the FMHE compensation unit is mounted on the wrist of a robotic manipulator driven by a digital servomotor to enable multiaxis compliance (Fig. 4B). When the manipulator faces an obstacle during operation, this unit can be bent to compensate for angular movement and prevent the damage of servomotor due to excessive torque. At room temperature, the torque of the servomotor increases linearly up to its maximum value with the bending of the FMHE unit, showing a maximum compensation angle of 7.3° (Fig. 4E). With a 5-V dc power supply, the FMHE softens at the critical bending angle to significantly reduce the stiffness of the device, which further increases the bending angle to 16.5° to compensate more angular movement (inset in Fig. 4F). We also coat a thermochromic elastomer layer on the FMHE_3_ columns in the compensation unit to indicate its high/low stiffness state by different colors (Fig. 4G). See text S4 for experimental details in Fig. 4 (E to G).

Compared to state-of-the-art rubber-based compensators, our FMHE-based units show an order of magnitude improved capability (bending angle of 16.5° over 1.1° of existing commercial systems, e.g., Schunk FUS unit). To illustrate this advantage, we demonstrate that the FMHE compensator can prevent the manipulator from damage in complex operational environments (see fig. S20 and movies S3 and S4 for details). Critically, in addition to bending, our design can also compensate for compressive and torsional movements. Such a multiaxis unit with self-adjusting stiffness and a large compensation range can bring unprecedented flexibility to robotic manipulators, greatly reducing hardware and software costs.

**Fig. 5.**
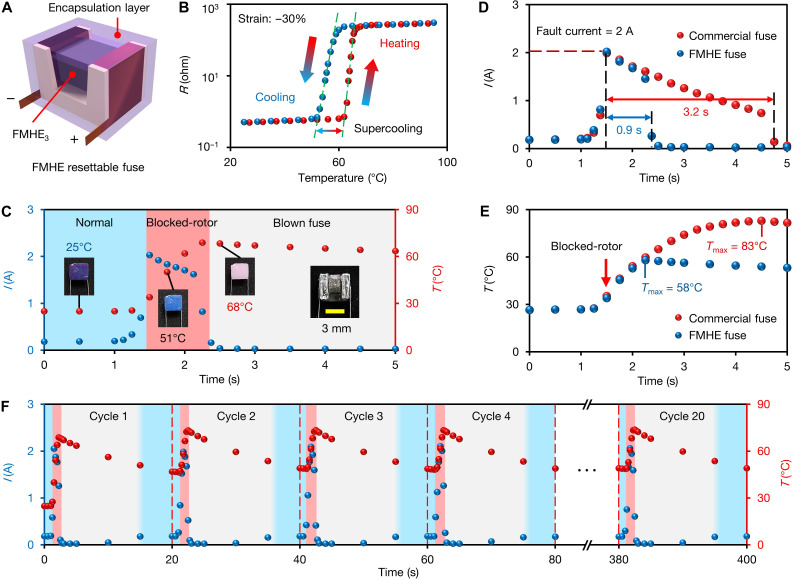
Resettable current-limiting fuse based on the FMHE_3_. (**A**) Schematic diagram of the FMHE resettable fuse. (**B**) Resistance-temperature curve of the FMHE fuse in a heating-cooling cycle. (**C**) Current-time and temperature-time curves of the FMHE fuse during operation/after the blocking of the servomotor. The fourth inset shows a photo of a FMHE fuse without the encapsulation layer. (**D**) Current-time and (**E**) casing temperature-time curves of the servomotor equipped with the FMHE fuse and a commercial PPTC fuse after the servomotor is blocked. (**F**) Current and temperature versus time plots of the FMHE fuse during a cyclic fuse blow test. The blue, red, and gray shaded regions in (C) and (F) correspond to the normal operation, blocked-rotor, and blown fuse states, respectively.

In our second demonstration, we present a highly compact, resettable current-liming fuse based on the FMHE by harnessing its variable resistance effect. In many electronic devices, electrical current rapidly increases in case of a short-circuit or component fault, resulting in overheating and damage to the device. Traditional current-limiting fuses are usually not reusable. State-of-the-art resettable fuses, such as dielectrophoresis-operated fuses (*42*), liquid metal fuses based on the pinch effect (*43*), or polymeric positive temperature coefficient (PPTC) fuses (*44*), have very limited application scopes because of their complex structure, large size (>10 mm), or long response time (>1 s). Our FMHE fuse design addresses all these drawbacks. As shown in Fig. 5A, the main structure of the fuse is a 3D printed polycarbonate holder with a trapezoidal groove that attaches two copper electrodes on both sides. An FMHE_3_ block larger than the trapezoidal groove is then squeezed into the groove with adjustable compressive strain to achieve different initial resistances and adjustable fusing currents from 0.1 to 10 A. Last, the device is encapsulated by a layer of thermochromic elastomer to enhance the structural durability and visually indicate the working state.

We use a servomotor as a case study—an FMHE fuse (2 mm by 3 mm by 3 mm) is connected in series with the servomotor with a circuit voltage of 6 V. We set the compressive strain of the FMHE_3_ block at 25% to adjust the fusing current to ~1 A. Before use, we heat and cool the FMHE fuse for three cycles to achieve stable resistance-temperature behavior (see Fig. 5B). Its resistance is 0.51 ohm at 25°C and reversibly increases to 255 ohm when heated to 70°C. Figure 5C shows the current passing through the FMHE fuse and its temperature change over time. At first, the servomotor works normally with a circuit current of 0.17 A. At 1.5 s, the servomotor is manually blocked from rotating, raising the current to 2 A. The FMHE fuse then rapidly heats up at an initial rate of ~60°C·s^−1^ because of its low volumetric heat capacity (2.06 J·cm^−3^·K^−1^) and high heating power (2 W) (see calculation in text S5 and table S1). The color of the thermochromic encapsulation layer changes to blue to warn the user. After reaching 69°C at 2.3 s, the fuse resistance rapidly increases by 500 times. The encapsulation layer turns white, indicating the blown fuse state, while the corresponding current drops rapidly to 23 mA to protect the servomotor. When the FMHE fuse cools back to <50°C, the encapsulation layer turns purple, indicating that it can be used again.

Figure 5 (D and E) compares the current/temperature change of the blocked servomotor equipped with the FMHE fuse and a commercial PPTC-resettable fuse (see details in Materials and Methods). The commercial resettable fuse takes 3.2 s to blow. The casing temperature of the servomotor rises to 83°C during this period, causing irreversible damage. When using the FMHE fuse, the current is limited to <100 mA in 0.9 s after the fault, limiting the casing temperature to 58°C. Such a quick response effectively protects the servomotor from any damage.

To demonstrate the reusability of the FMHE fuse, we carry out a 20-cycle fuse blow test and show its current-time and temperature-time curves in Fig. 5F. In each cycle, the FMHE fuse is blown within 1 s after the servomotor is blocked. It cools to 50°C under ambient temperature in ~14 s, with its resistance recovering to ~0.6 ohm to reconnect the circuit (corresponding to the blue shaded region in Fig. 5F). This cyclic test shows the fast response and recovery speed of the FMHE-resettable fuse and its excellent cyclic stability.

By reducing the size, our FMHE fuse can achieve a much faster response. To show its potential, we present a smaller FMHE fuse (1 mm by 1 mm by 1 mm) with a response time of only 85 ms. In terms of volume, this device is an order of magnitude smaller but is >10 times faster than commercial and other reported resettable fuses (see fig. S21 for details). Crucially, the unique mechano-responsive property of the FMHE fuse enables a wide range of adjustable fusing currents on the same device, offering vast versatility and functionality that has never been possible before.

## DISCUSSION

In summary, we have created a type of electro-mechano responsive FMHE that exhibits variable electromechanical properties. Through experiments and numerical simulations, we demonstrate its unconventional negative piezoresistivity, tunable resistivity, strain sensitivity, and stiffness. By combining the high strain sensitivity and variable resistance/stiffness of the FMHE, we develop a variable stiffness compensation unit that can deform to protect a robotic manipulator from excessive compressive, bending, and torsional movements. The unit can adjust its stiffness under deformations without external control, offering an order of magnitude wider protection range than a state-of-the-art one. To underscore the broad applicability of our design, we also demonstrate an FMHE resettable fuse that offers adjustable fusing currents while significantly outperforming state-of-the-art solutions in terms of compactness, range of operating current, and response speed. This material family shows great potential in next-generation intelligent and resilient robotics and electronics.

## MATERIALS AND METHODS

### Materials

Indium (99.995%), bismuth (99.99%), tin (99.95%), and Ga (99.99%) were purchased from Magnametals, UK. The spiked Ni microparticles (2 to 5 μm in diameter) were purchased from APC Pure, UK. The Ecoflex 00-30 addition cure silicone rubber was purchased from Smooth-On Inc., USA. The SYLGARD 184 Silicone Elastomer Curing Agent and SYLGARD 184 Silicone Elastomer Base were purchased from Dow Corning, USA. Thermochromic powders were purchased from Guangzhou CHONGYU Industrial Materials Technology Ltd., China.

### Preparation of the FMHE

To fabricate FMHE samples, we first prepared the FM alloy. We cut indium (51 wt %), bismuth (32.5 wt %), and tin (16.5 wt %) into pieces less than 3 mm in size and placed them in a glass beaker on a heating plate to melt at 220°C for 1 hour. Then, we stirred the melted metal with a glass rod and continued heating it for 5 min. After cooling at ambient for 5 min, the liquid alloy was poured into a deionized water tank to obtain an FM alloy block. When preparing FMHEs, we first mixed 1 wt % of Ga into the melted FM at 100°C to prepare Ga-doped FM alloy (its melting point is ~60°C). Then, we placed it and PDMS in a glass beaker placed in an 80°C water bath with a volume ratio of 1:1 (PDMS is a mixture of 90 wt % of SYLGARD 184 silicone elastomer base and 10 wt % of SYLGARD 184 silicone elastomer curing agent). They were mixed using a high-speed electric stirrer (rotating speed of 300 rpm) for 5 min. The electric stirrer was equipped with a flat mixing stick with a cross section of 2 mm by 5 mm. We then mixed the obtained FM-PDMS dispersion with Ni microparticles for 4 min at a rotating speed of 200 rpm (for the FMHE_0_ without Ni particles, this step was skipped). After that, the mixture was degassed in a vacuum chamber for 20 min to remove air bubbles. Last, it was poured into 3D printed polyurethane molds and cured at 75°C for 12 hours to obtain the FMHE samples. The volume ratio of PDMS/FM/Ni in FMHE_3_ is 1:1:0.34. We prepared cubic samples (8 mm by 8 mm by 8 mm) for compression and bar-shaped samples (2 mm by 5 mm by 30 mm) for stretching.

### Preparation of the thermochromic elastomer

The thermochromic elastomer is Ecoflex filled with thermochromic powder. We used Ecoflex as the matrix because of its high stretchability and compliance. The thermochromic powder is composed of spherical particles with a diameter of 2 to 7 μm. Its color changes when the temperature rises above the activation temperature and reversibly changes back to its original color when the temperature recovers. The thermochromic powder used in this work is purple below 49°C, blue between 49° and 60°C, and white above 60°C. We mixed the thermochromic powder into Ecoflex with a mass fraction of 2.5% and placed the mixture in a vacuum chamber for 20 min for degassing. Ecoflex is a mixture of Ecoflex part A and part B with a mass ratio of 1:1. The FMHE column or FMHE fuse was immersed in a mold filled with the mixture and cured at ambient for 3 hours to form a thermochromic Ecoflex encapsulation layer on the surface. The thickness of the thermochromic encapsulation layer was about 0.5 mm.

### Experimental equipment and tools

A Gemini SEM 500 field emission scanning electron microscope was used to obtain the SEM and EDS images. An Ultimaker S5 3D printer was used to print the polyurethane molds for curing the FMHE samples and the polycarbonate shell of the FMHE fuse. A Single Column Mechanical Testing Machine (YK-Y0026, Dongguan Yaoke Instrument Equipment Co. Ltd., China) was used to compress or stretch the block or bar FMHE samples at a speed of 5% min^−1^ (3% min^−1^ in Fig. 3, A and B) to measure their resistance/stress-strain curves. When heating the sample from the outside (Figs. 2 and 3, A and B), the block samples were heated using a Thermoelectric Peltier chip (TEC1-12706), and the bar samples were heated using an induction heater (dc: 5 V, 12 A). In Fig. 2, to avoid the hysteresis of the sample temperature increase relative to the chip temperature increase, we increased the chip temperature by about 1°C for each measurement and waited for 10 s after the temperature stabilized to measure the sample resistance. A FLUKE 8845A digital multimeter with a resistance range of 100 megohms was used to measure the resistance and current. The final resistance is the measured resistance minus the internal resistance of the circuit with no sample present. A DSSrevo coreless digital servo (DS3230) was used to drive the variable stiffness manipulator. A Voltcraft temperature-measuring instrument (PL-125-T2USB) was used to record temperature data. When measuring the temperature, the thermocouple probe was attached to the surface of the FMHE sample or measured object. A CAT S60 FLIR infrared thermal camera was used to measure the temperature of the FMHE compensation unit, the FMHE fuse, and the servomotor. A Littelfuse 1206L050 resettable PPTC fuse (maximum voltage: 6 V; trip current: 1.0 A) was used to compare with the FMHE fuse. COMSOL Multiphysics 5.2 software package (Burlington, MA, USA) is used to simulate and calculate the deformation and resistance of the FMHE during deformation at different temperatures.

## Supplementary Materials

### This PDF file includes:

Text S1 to S5 Figs. S1 to S21 Table S1 References
