# Supplementary material for: Electro-mechano responsive elastomers with self-tunable conductivity and stiffness
Source: Sci Adv. 2023 Jan 25;9(4):eadf1141. doi: 10.1126/sciadv.adf1141 (PMC9876544; doi:10.1126/sciadv.adf1141)
Supplement: Supplementary file 1 — Text S1 to S5 Figs. S1 to S21 Table S1 References [file sciadv.adf1141_sm.pdf]

Science Advances
AAAS

Supplementary Materials for

Electro-mechano responsive elastomers with self-tunable conductivity and stiffness

Guolin Yun *et al.*

Corresponding author: Weihua Li, weihuali@uow.edu.au; Shiwu Zhang, swzhang@ustc.edu.cn; Shi-Yang Tang, s.tang@bham.ac.uk

Sci. Adv. **9**, eadf1141 (2023)
DOI: 10.1126/sciadv.adf1141

The PDF file includes:

Text S1 to S5
Figs. S1 to S21
Table S1
Legends for movies S1 to S4
References

Other Supplementary Material for this manuscript includes the following:

Movies S1 to S4

Supplementary Text

Supplementary Text 1. Reasons for the resistance increase of FMHE samples during heating.

Due to the significant thermal expansion of polymer matrices, most conductive elastomers exhibit a positive temperature coefficient, that is, their resistance increases with temperature. The thermal expansion rate of PDMS is 0.034% $°C^{-1}$(*37*). As a result, the $FMHE_3$ samples in our work expand by around 0.8% when heated from 25 °C to 60 °C. In metal-polymer conductive composites, their resistance depends on the contact resistance between metal microparticles, i.e., the electron tunneling probability between them, which is extremely sensitive to particle spacing(*31*). Therefore, even an expansion of 0.8% can also lead to the separation of metal particles in the FMHE and a significant increase (>20%) in electrical resistance. Our experiments show that when the $FMHE_3$ sample is placed in a copper mold to prevent its expansion, its resistance increases by only <3% when heated to 60 °C. This proves that the resistance increase of FMHE during heating mainly corresponds to the expansion of PDMS. Furthermore, the electrical resistance of metal fillers in FMHE increases by about 14% when heated to 60 °C due to their positive temperature coefficient of ~0.004/°C. However, the resistance of FMHE mainly depends on the contact resistance between the conductive particles rather than the very low resistance of the metal particles themselves. Therefore, this is not the main factor affecting the resistance change of FMHE during heating.

Supplementary Text 2. Factors affecting the timing and speed of stiffness reduction of the FMHE.

According to Fig. 3, F to H, when compressing FMHE samples under an external voltage, the voltage and compression speed will affect the critical strain of stiffness change and the decline speed of the stress curve of FMHE samples. A higher voltage can reduce the required strain for

melting FM, as reflected by the earlier softening indicated by the red curve given in Fig. 3F and the lower critical strain shown in Fig. 3H. A fast compression speed will accelerate the stiffness change process. Due to the lag of the temperature rise of the sample relative to its resistance increase, a faster compression speed can lead to a higher current and heating power before FM starts melting, leading to a higher temperature of FMHE samples. This is also demonstrated by the comparison of maximum temperatures at different voltages and compression speeds; see fig. S18. The maximum temperature of the sample compressed at 6% min^{-1} is 69.3 °C, while that of the sample compressed at 12% min^{-1} is 80.7 °C. Higher temperatures can melt FM particles faster, resulting in a rapid reduction in stiffness, as indicated by the rapidly decreasing red stress curve given in Fig. 3F.

Supplementary Text 3. Variable stiffness function of the FMHE variable stiffness compensation unit.

The FMHE self-triggered variable stiffness compensation unit can realise compression, bending and torsion with variable and adjustable stiffness. Its variable stiffness mechanical load curves during compression (speed of 0.4 $mm\cdot min^{-1}$) and bending (speed of 2° min^{-1}) are given in Fig. 4, C and D. At room temperature, its compression force basically increases linearly with compression distance to 286 N at 1.5 mm, with a stiffness coefficient of 191 $kN\cdot m^{-1}$. At 80 °C, the force at 1.5 mm drops to 130 N, showing a lower stiffness coefficient of 86.7 $kN\cdot m^{-1}$. To demonstrate its self-triggered variable stiffness function, we apply a fixed voltage of 5 V to it during compression. The initial resistance of the compensation unit is ~100 Ω. As the force increases to 83 N at 0.6 mm, its resistance drops to 2.5 Ω, and its temperature reaches 60 °C. Its stiffness then decreases with the melting of FM, as shown in the yellow force–distance curve in

Fig. 4C. As the compression distance increases from 0.7 mm to 1.5 mm, the compensation unit maintains a temperature of ~75 °C and a low stiffness coefficient of 44 $kN \cdot m^{-1}$. For the case of bending, the compensation unit also shows a similar variable stiffness phenomenon. It has bending stiffness coefficients of 0.413 N·m/° and 0.193 N·m/° at 25 °C and 80 °C, respectively. When powered by a 5 V supply during bending, its temperature rises to 75 °C at a bending degree of 3.7°. After that, its bending moment increases slightly, showing a bending stiffness close to 0. After the bending angle reached 7.3°, its bending moment curve intersects and overlaps with the curve at 80 °C.

Supplementary Text 4. Demonstration of the FMHE self-triggered compensation unit in the manipulator.

In the servo motor-driven manipulator equipped with a variable stiffness compensation unit, the axis of the servo motor is perpendicular to the compensation unit axis. Therefore, the manipulator swung back and forth like a pendulum when the servo motor is activated. A manipulator without the FMHE unit will get stuck when encountering obstacles during its swing, causing an immediate blocked rotor and damage to the servo motor. This will lead to high internal mechanical stress and overheating of the servo motor, which will cause damage. After being equipped with the flexible FMHE compensation unit, this problem can be solved. When no voltage is applied to the compensation unit, the torque–time and current–time curves of the servo motor after the manipulator is stuck are shown in Fig. 4E. The servo motor rotates at a constant speed of $0.54° \cdot s^{-1}$ with an initial working current of 0.17 A at 0 s. Since the temperature of the FMHE unit remains unchanged, its bending stiffness is constant at 0.413 N·m/°. With the rotation of the servo motor, the bending angle and moment of the FMHE unit increase, resulting in an increase in the

torque and current of the servo motor. At 13.5 s, its torque reaches the blocked-rotor torque of 3 N·m, and the current increases to 2 A, showing a bending compensation angle of 7.3°. When powered by a 5 V supply, the compensation unit softens at a bending angle of 4.9°, delaying the time for the servo motor to reach the blocked-rotor torque. This thereby further increases the bending compensation angle of the compensation unit to 16.5° (see Fig. 4F). The thermochromic elastomer encapsulation layer on the $FMHE_3$ columns can indicate the working state of the compensation unit. The colours of the encapsulation layer at < 49 °C, 49~60 °C and > 60 °C are purple, blue, and white, respectively. From Fig. 4, F and G, the initial temperature of the FMHE unit is 26 °C with a purple encapsulation layer. At 6 s, the encapsulation layer turns blue, indicating that the FMHE columns have been heated and FM is about to melt. From 7 s to 16 s, the torque of the servo motor is basically unchanged, corresponding to the 0-stiffness section of the yellow bending moment curve given in Fig. 4D. After that, the torque increases slowly with the angle of the servo motor, and the encapsulation layer remains white, indicating that the FMHE unit is in the low stiffness state. At 30.5 s, the servo motor stops rotating at a blocked-rotor angle of 16.5°.

Supplementary Text 5. Calculation of the volumetric heat capacity of $FMHE_3$ and heating rate of the FMHE fuse.

The volumetric heat capacity describes the ability of a unit volume of a material to store internal energy when undergoing a given temperature change (without undergoing a phase transition). It is the product of the material density and the specific heat capacity. The volumetric heat capacity of the FMHE is equal to the volume-weighted average of the volumetric heat capacities of the individual components. The volume ratio of PDMS, Ni particles and FM in

$FMHE_3$ is 1:0.337:1. According to the density and specific heat capacity of each material in the following table, we calculate the volumetric heat capacity of $FMHE_3$, which is 2.06 $J·cm^{-3}·K^{-1}$.

Then we can calculate the heating rate of the FMHE fuse when the servo motor is blocked. In Fig. 5C, the current passing through the FMHE fuse is 2.0 A after blocking. Since the resistance of the FMHE fuse is 0.51 Ω, its initial heating power will be 2.04 W ($P = R·I^2$). According to the size of the FMHE block in the FMHE fuse (2×2×3 mm), it will be heated at a theoretical rate of ~82.5 $°C·s^{-1}$ (2.04 W/[(0.012 cm^3)·(2.06 $J·cm^{-3}·K^{-1}$)]). Due to the heat dissipation through the copper electrodes and current reduction, the actual heating rate between 1.5 s and 2 s is about 50 $°C·s^{-1}$ (Fig. 5C).

Table S1.

Density, specific heat capacity, and volumetric heat capacity of raw materials for $FMHE_3$.

	PDMS	Ni	In	Bi	Sn	Ga	Field's metal
density ($g·cm^{-3}$)	1	8.9	7.3	9.8	7.28	5.9	7.90
specific heat capacity ($J·g^{-1}·K^{-1}$)	1.46	0.447	0.23	0.3	0.228	0.37	0.255
volumetric heat capacity ($J·cm^{-3}·K^{-1}$)	1.46	3.98	1.679	2.94	1.66	2.18	2.01

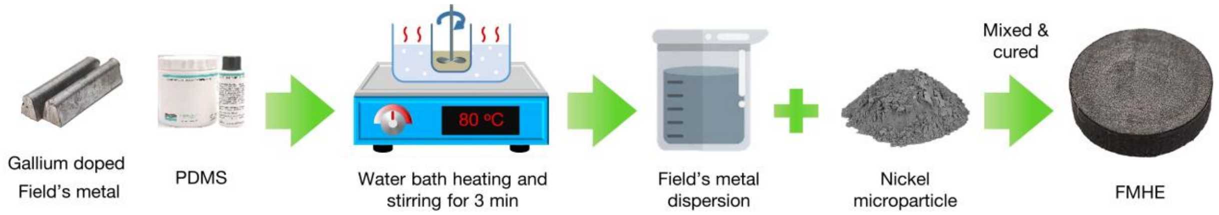

Fig. S1.

Procedure for preparing the FMHE.

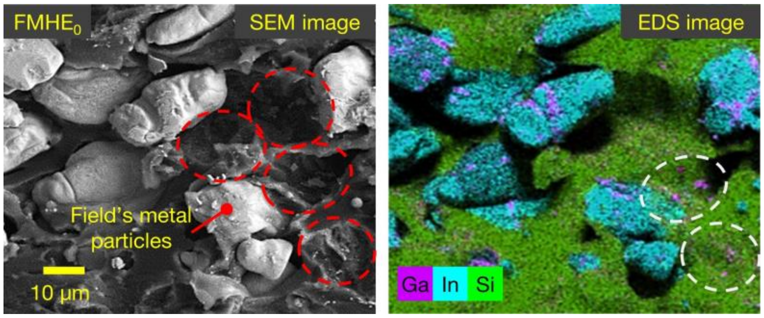

Fig. S2.

Scanning electron microscopy (SEM) and energy dispersive spectroscopy (EDS) images of the cross-section of $FMHE_0$. The pits in the red circles are formed by the removal of FM particles.

Figure S2 shows the scanning electron microscopy (SEM) and energy-dispersive X-ray spectroscopy (EDS) images of the cross-section of $FMHE_0$ samples. The diameter of the FM particles in the $FMHE_0$ is 15~30 μm. Due to their high (50%) volume fraction, these FM particles are in close contact with one another with nanometre-wide gaps in between. The EDS image shows the elemental distribution of gallium, indium and silicon in $FMHE_0$. The distribution area of gallium overlaps with that of indium, showing the gallium oxide layer on the surface of the FM particles (also visible in SEM images). Gallium oxide usually forms on the surface of gallium-based liquid metals. For the gallium-doped FM in this work, the formation of the gallium oxide layer is due to the fact that this can provide the lowest Gibbs free energy of the FM droplet (ref *33* in the main manuscript).

We note that FM particles also exist in the red dotted circles, but they are attached to the other cross-section of the sample after fracture, as evidenced by the pits in the red circles in the SEM image. In addition, the residual Ga oxide skin in the white circles in the EDS image corresponds to the pits in the SEM image, which further proves the existence of FM particles in the circles.

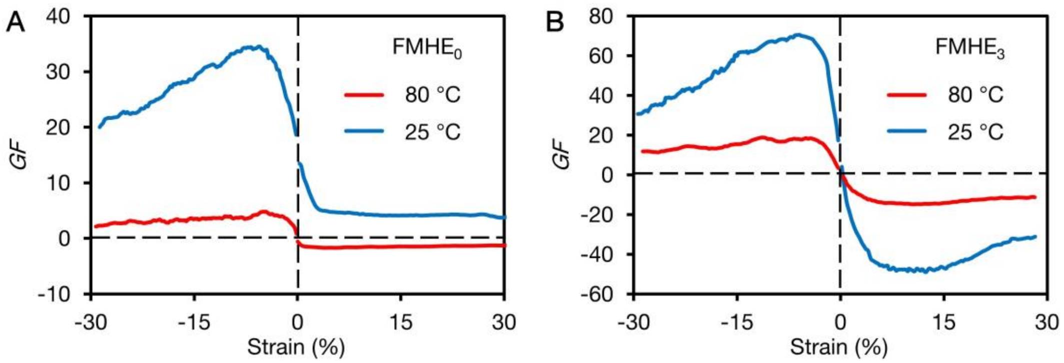

Fig. S3.

Gauge factor–strain curves of (A) $FMHE_0$ and (B) $FMHE_3$ at 25 °C and 80 °C.

We calculate the gauge factor ($GF = \Delta\rho/\rho\varepsilon$, where ρ and ε are the electrical resistivity and strain, respectively) of $FMHE_0$ and $FMHE_3$ at 25 °C and 80 °C, as shown in Fig. S3. The *GF* quantifies the strain sensitivity and piezoresistive effect of the FMHE, with a higher value indicating higher sensitivity. A positive *GF* indicates a positive piezoresistive effect. At 25 °C, $FMHE_0$ shows a maximum *GF* of 34.1 at 5% compressive strain. In contrast, $FMHE_3$ exhibits a higher strain sensitivity with a maximum *GF* of 70.5. In addition, the negative *GF* of $FMHE_3$ during stretching also shows its special negative piezoresistive effect. At 80 °C, the melting of FM significantly reduces the strain sensitivity of FMHEs, as indicated by their low *GF*. The *GFs* of $FMHE_0$ during compression and stretching are only 3.6 and −1.6, respectively. Unlike $FMHE_0$, the strain sensitivity of $FMHE_3$ also drops sharply after FM melts but remains high. Its GF under 5% compressive and tensile strain is 18.4 and −13.4, respectively, which is much higher than that of $FMHE_0$.

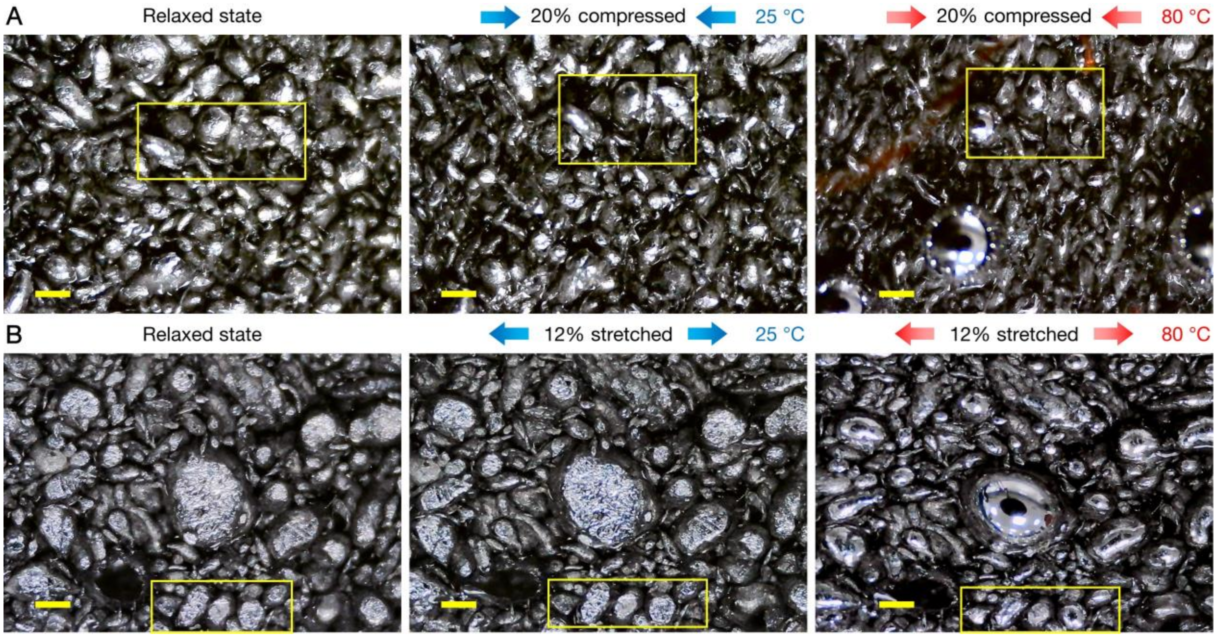

Fig. S4.

Optical microscope images of the surfaces of $FMHE_0$ samples showing the space change between FM particles and their deformation upon melting under (A) compression and (B) stretching. Scale bars are 20 μm.

The FM particles in the yellow box show their close contact under compression and separation under stretching at 25 °C. As the deformed sample is heated to 80 °C, the FM particles melt and deform under stress, which is also clearly demonstrated in Movies S1 and S2. Note that some of the FM particles in the compressed sample are exposed to the air on the cross section. Therefore, they are forced out of the sample surface by pressure after melting, resulting in a leakage.

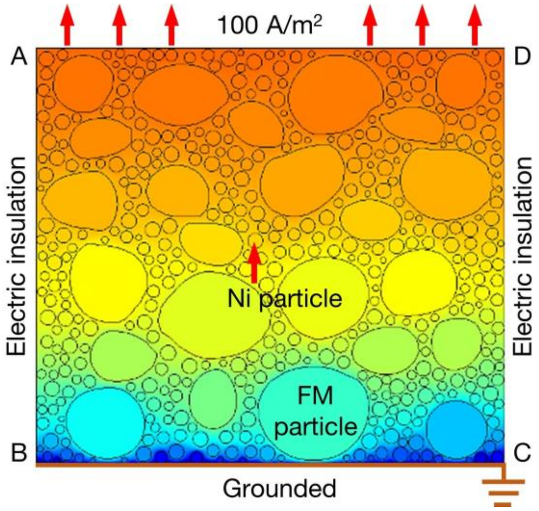

Fig. S5.

2D model of the FMHE used for the numerical simulation.

We use COMSOL finite element software to simulate the resistivity of the $FMHE_0$ and $FMHE_3$ 2D models (150×150 μm) during a compression process at 25 °C and 80 °C. The thickness of the model is set at 1 μm. The models are established based on the SEM images. The sizes of the Ni (2~5 μm) and FM (15~30 μm) particles are equal to those in the actual composites. We use an electrical-mechanical-thermal coupled multifield to simulate the mechanical deformation and resistivity of the model. The material parameters of the fillers and PDMS matrix (mainly including the elastic modulus, Poisson's ratio, conductivity, dielectric constant, and coefficient of thermal expansion) are consistent with the COMSOL material library. The FM uses material parameters corresponding to the solid or liquid state at 25 °C or 80 °C, respectively. In the simulation model, we omit the gallium oxide layer on the surface of the melted FM droplet. This is because the thickness of this gallium oxide film is only 1~3 nm (ref *34* in the main manuscript), which is much smaller than the diameter of FM droplets (10~20 μm). Such a thin oxide layer does not affect the

mechanical deformation of the FM droplets. Additionally, since the conductivity of both the FM droplet and the gallium oxide is much higher than that of the PDMS matrix, this extremely thin oxide layer also does not affect the simulation for the resistivity of the composite. The mechanical deformation and microstructure change of the model are simulated by applying a fixed displacement load to the upper side of the model. Figure S5 shows the boundary conditions in the simulation. The left and right sides of the 2D model are electrically insulated, the lower side is grounded, and the current density through the upper side is fixed at 100 $A \cdot m^{-2}$. The resistivity of the FMHE model can be calculated based on the drop in electrical potential.

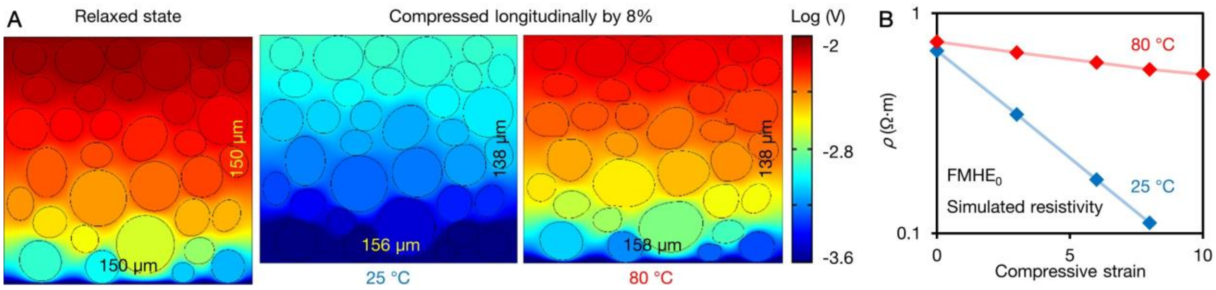

Fig. S6.

Numerical simulation of the resistivity of FMHE$_0$. (**A**) Simulation results of the electrical potential distribution of FMHE$_0$ at different strains and temperatures. (**B**) Numerical simulated resistivity–strain curves of FMHE$_0$ at 25 °C and 80 °C.

Figure S6A shows the microstructure and electrical potential distribution of FMHE$_0$ compressed at 25 °C and 80 °C. The potential drops when FMHE$_0$ is compressed by 8% at 80 °C is similar to that in the relaxed state, reflecting the relatively stable resistivity when FM melts. In contrast, the voltage drops at 25 °C are very small, corresponding to low resistivity and high strain sensitivity. The resistivity change reflected in the simulated resistivity–strain curves given in Fig. S6B is also consistent with the experimental results.

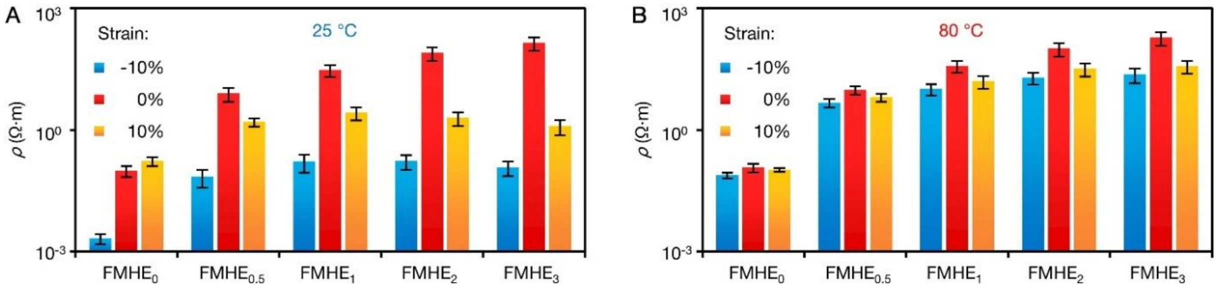

Fig. S7.

Resistivity of the FMHE with different Ni contents at (A) 25 °C and (B) 80 °C. The values of the error bars are the standard deviation of the sample resistivity under 5 measurements.

To investigate the influence of Ni content on the electrical properties of FMHE, we compare the resistivity of $FMHE_{0/0.5/1/2/3}$ samples at 25 °C and 80 °C. From Fig. S7A, the initial resistivity and strain sensitivity of the FMHE increase significantly with increasing Ni content. In addition, the resistivity of $FMHE_{0.5}$ decreases by 80% when stretched by 10%, proving that FMHE can exhibit a negative piezoresistivity during stretching even with only a small amount of Ni particles. However, Ni particles will also slightly reduce the stability of the FMHE resistivity, as indicated by the relatively large error bars in Fig. S7A. Figure S7B compares the resistivity of $FMHE_{0/0.5/1/2/3}$ samples at strains of −10%, 0% and 10% when the FM particles melt. We can see that the melting of FM particles shows little influence on the initial resistivity of the FMHE samples but greatly increases their resistivity under mechanical deformation (i.e., reduces their strain sensitivity). When compressed by 10% at 80 °C, the conductivity of $FMHE_{0/0.5/1/2/3}$ increases by 0.6, 1.1, 2.7, 4.2, and 7 times, respectively.

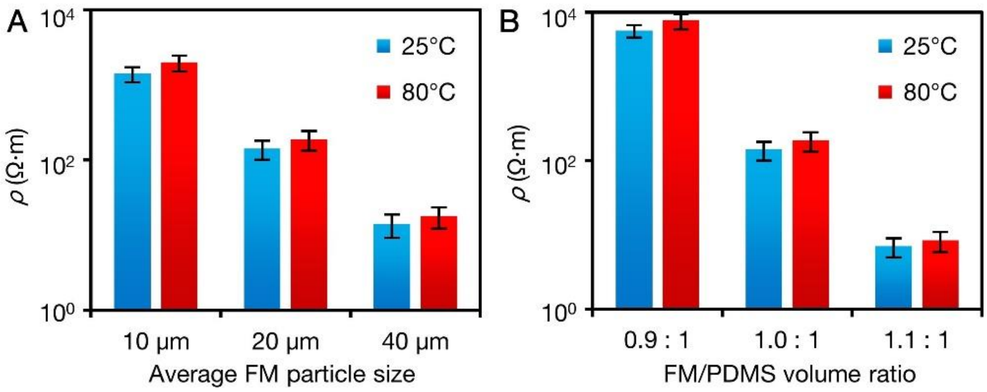

Fig. S8.

Resistivity of $FMHE_3$ samples with different FM (A) particle sizes and (B) contents at 25 °C and 80 °C. The values of the error bars are the standard deviation of the sample resistivity under 5 measurements.

To study the influence of the particle size and volume fraction of FM on the resistivity of FMHE, we prepare a set of $FMHE_3$ samples with different average FM particle sizes and FM contents and compare their initial resistivities at 25 °C and 80 °C. According to Fig. S8A, the resistivity of $FMHE_3$ will decrease by >10 times when the average FM particle size is doubled. However, the large particle size also leads to lower resistivity stability. The FM content also has a significant influence on the electrical conductivity of FMHE. From Fig. S8B, when the volume of FM increases by 10%, the resistivity of $FMHE_3$ can be reduced by approximately 30 times. For all $FMHE_3$ samples, the melting of FM particles increases their resistivity by 20~40%.

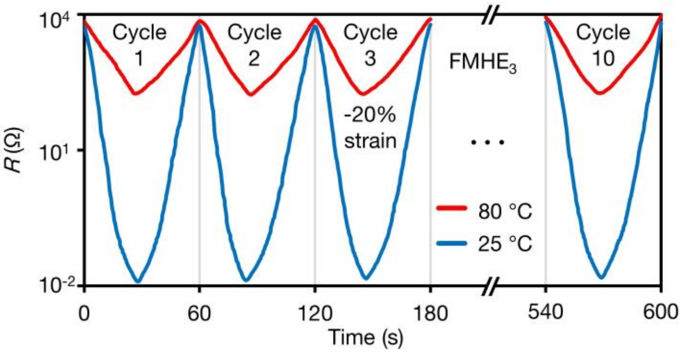

Fig. S9.

Resistance–time curves of $FMHE_3$ over 10 cycles during cyclic loading tests at 25 °C and 80 °C.

We conduct 10 cycles of cyclic compression tests on $FMHE_3$ samples at 25 °C and 80 °C with a maximum compressive strain of 20%. Figure S9 gives the resistance–time curves for the first three and last cycles. In each compression cycle, the resistance of $FMHE_3$ decreases steadily and basically returns to the initial value. After 10 cycles, the initial resistance increases by approximately 10%, showing good stability.

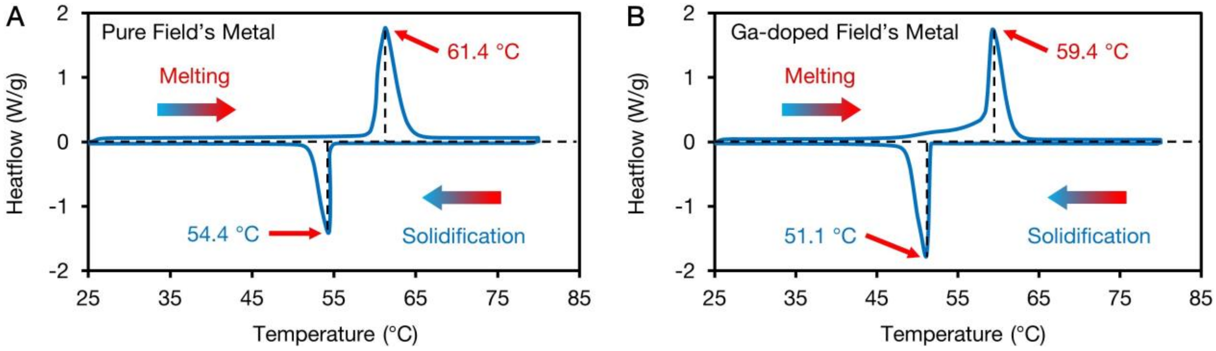

Fig. S10.

Differential scanning calorimetry (DSC) curve of the (A) pure FM alloy and (B) Ga (1 wt%)-doped FM alloy.

According to the DSC curve, the melting and freezing points of pure FM are 61.4 and 54.4 °C, respectively, showing a supercooling degree of 7 °C. As a comparison, the melting and freezing points of Ga-doped FM are 59.4 and 51.1 °C, respectively, showing a lower phase transition temperature and a more pronounced supercooling effect (supercooling degree of 8.3 °C).

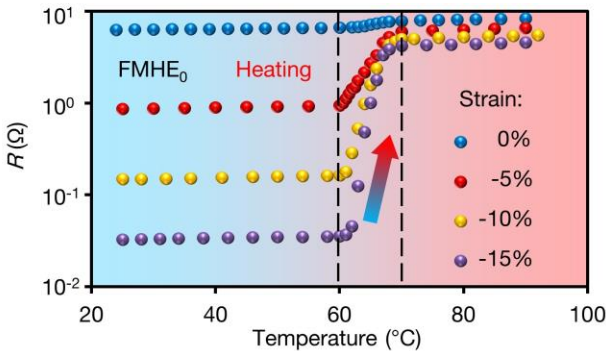

Fig. S11.

Resistance–temperature curves of the $FMHE_0$ sample under different strains during heating.

Under different compressive strains, the resistance of $FMHE_0$ increases sharply during the melting of FM particles from 60 °C to 70 °C and increases slightly with temperature when the FM is solid or liquid.

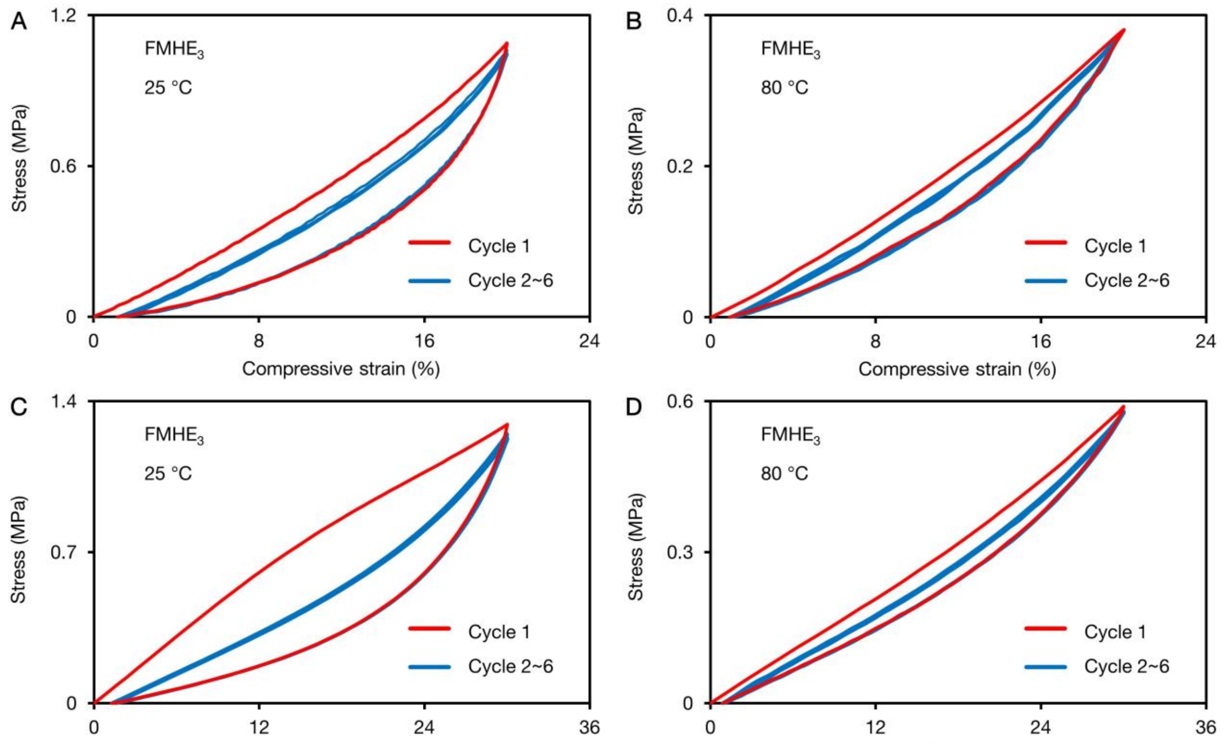

Fig. S12.

Cyclic stress–strain curves of $FMHE_3$ under different conditions of (A) compression at 25 °C; (B) compression at 80 °C; (C) stretching at 25 °C; and (D) stretching at 80 °C.

To study the mechanical stability of $FMHE_3$ under cyclic loads, we perform cyclic compression/stretching experiments on $FMHE_3$ samples at 25 °C and 80 °C for 6 cycles. According to Fig. S12, for each condition, the sample shows more obvious elastic hysteresis in the first cycle (larger elastic hysteresis loop). Their stress curves closely overlap in the subsequent cycles, exhibiting good mechanical stability. After changing the temperature, the curves also overlap after the first cycle. In addition, when FM melts at 80 °C, the elastic hysteresis of $FMHE_3$ is significantly weakened, indicating lower energy dissipation. When loaded at 25 °C, the deformation of the filler network in the FMHE composite results in a position change of the non-

deformable solid FM particles. During this process, friction between solid particles as well as between particles and the polymer matrix results in significant energy losses (*39*). On the other hand, when loaded at 80 °C, the liquid FM droplets can deform along with the matrix with almost no frictional energy loss. In addition, the deformation of the FM droplet also relieves the actual strain in the polymer matrix, thereby reducing the internal friction loss of the matrix.

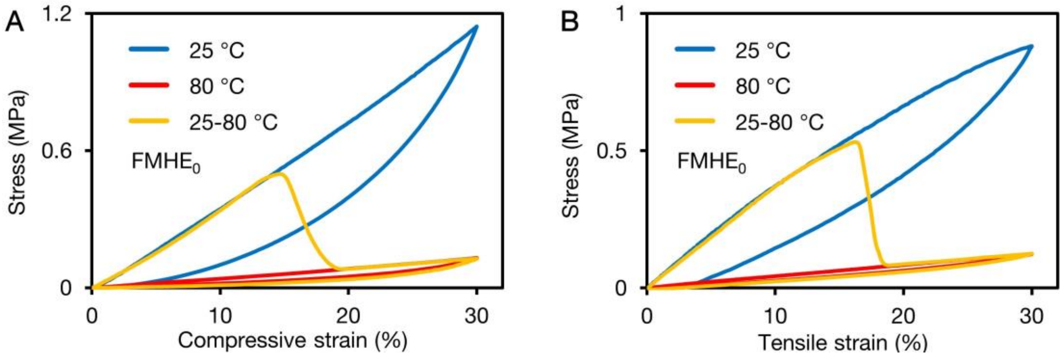

Fig. S13.

Stress–strain curves of $FMHE_0$ at different temperatures during (A) compression and (B) stretching.

Figure S13 shows an obvious variable stiffness effect of $FMHE_0$ under both compression and stretching. As the temperature increases from 25 °C to 80 °C, its stress at 30% compressive and tensile strain drops by 89.8% and 86.8%, respectively. When heated during compression/stretching, the stress curves before and after FM melting also overlap with its stress curves at 25 °C and 80 °C, respectively.

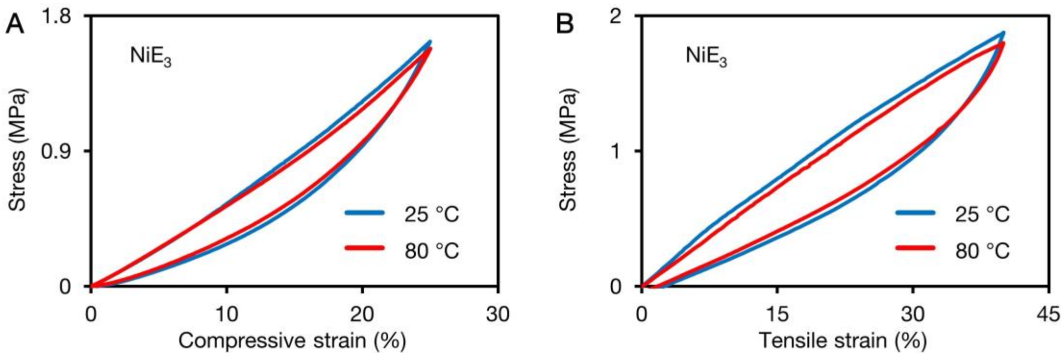

Fig. S14.

Stress–strain curves of NiE_3 at 25 °C and 80 °C during (A) compression and (B) stretching.

To verify that the variable stiffness effect of the FMHE results from the phase transition of FM when the temperature changes, we prepare Ni particle-filled elastomers without FM fillers, denoted as NiE_3. The mass ratio of Ni particles to the PDMS matrix of these samples is 3:1, which is consistent with that of $FMHE_3$. From Fig. S14, their stress–strain curves at 25 °C and 80 °C differ only slightly, indicating that the variable stiffness effect of FMHE is independent of the Ni particles and the PDMS matrix.

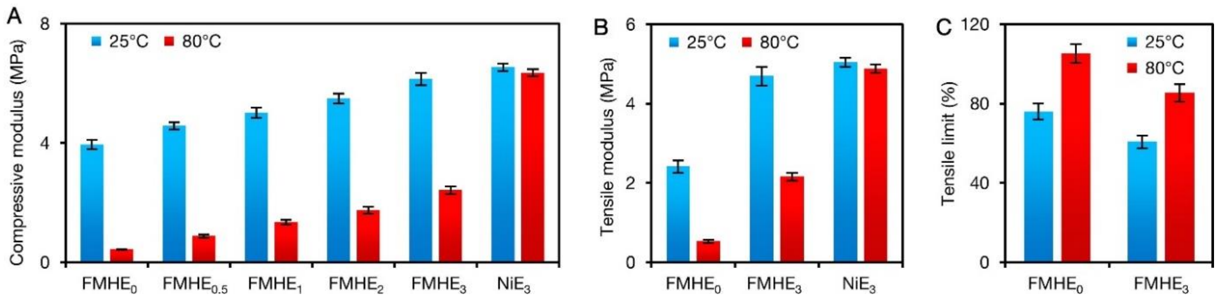

Fig. S15.

The (A) compressive modulus, (B) tensile modulus, and (C) tensile limit of the FMHE with different Ni contents at 25 °C and 80 °C. The values of the error bars are the standard deviation of the sample resistivity under 5 measurements.

To investigate the influence of Ni particles on the variable mechanical properties of the FMHE, we compare the elastic modulus and tensile limits of the FMHE with different Ni contents. The elastic modulus of the FMHE can be calculated from the slope of its stress–strain curve. Figure S15A shows the compressive modulus of $FMHE_{0/0.5/1/2/3}$ and NiE_3 at 25 °C and 80 °C. At room temperature, the compressive modulus of the FMHE increases with Ni content from 3.95 MPa ($FMHE_0$) to 6.14 MPa ($FMHE_3$) because of the increase in the solid filler volume fraction. When FM melts, the FMHE with a lower Ni content shows a more significant softening effect upon heating. For instance, the compressive modulus of $FMHE_0$ decreases by 9.5 times to only 418 kPa at 80 °C, which is even lower than that of the PDMS matrix we use (~1.0 MPa). In contrast, the compressive modulus of $FMHE_3$ decreases by only 61% when FM melts because Ni particles increase the stiffness of the FMHE at both low and high temperatures. For NiE_3, its compressive modulus at 25 °C and 80 °C differs by only 2.8%, which again proves that the variable stiffness effect of the FMHE is independent of the PDMS matrix and Ni particles. For the tensile modulus

shown in Fig. S15B, with increasing Ni content, the FMHE shows an increased modulus and weakened variable stiffness effect. The tensile modulus of $FMHE_0$ and $FMHE_3$ at 25 °C is 4.7 and 2.3 times that at 80 °C, respectively. Furthermore, the melting of FM can also improve the stretchability of the FMHE. From Fig. S15C, the $FMHE_0$ bar will break at an average tensile strain of 76% at room temperature. After FM melts, it can be stretched by up to 110%. With the increase in Ni content, the stretchability of the FMHE decreases but can still be improved at a high temperature. The average tensile limit of $FMHE_3$ is 60.8% at 25 °C and can increase to 85.4% at 80 °C.

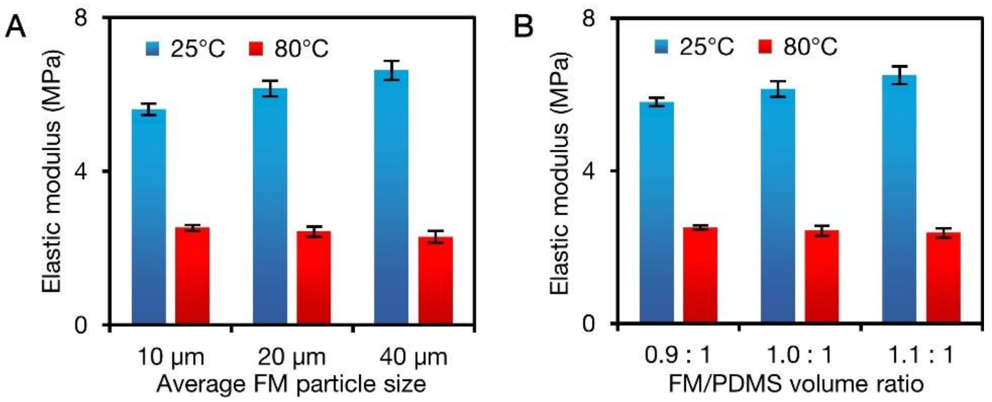

Fig. S16.

Elastic modulus of $FMHE_3$ samples with different FM (A) particle sizes and (B) contents at 25 °C and 80 °C. The values of the error bars are the standard deviation of the sample resistivity under 5 measurements.

To study the influence of the particle size and volume fraction of the FM on the elastic modulus of FMHE, we prepare a set of $FMHE_3$ samples with different average FM particle sizes and FM contents and compare their compressive moduli at 25 °C and 80 °C, as shown in Fig. S16. The elastic modulus of $FMHE_3$ increases with the FM particle size and content at 25 °C but shows the opposite trend at 80 °C. This means that $FMHE_3$ with a larger FM particle size and higher FM content has a more obvious variable stiffness effect.

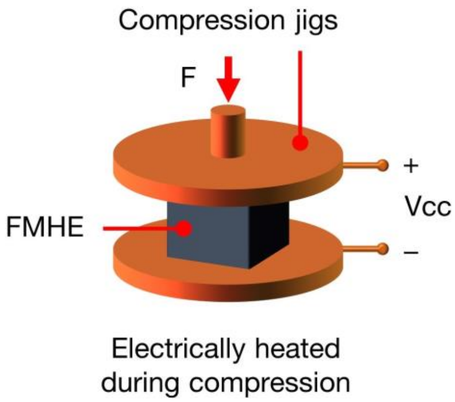

Fig. S17.

Experimental setup for applying a fixed voltage on an $FMHE_3$ sample during compression.

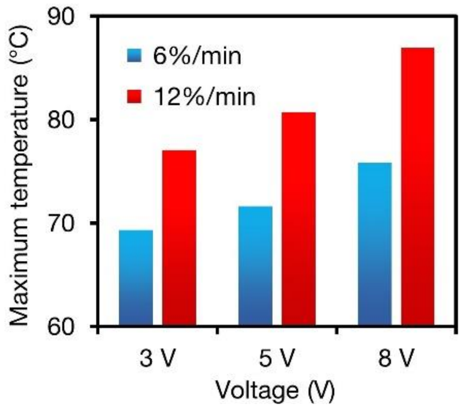

Fig. S18.

The maximum temperatures of $FMHE_3$ samples powered by a fixed voltage during compression with different voltages and compression speeds.

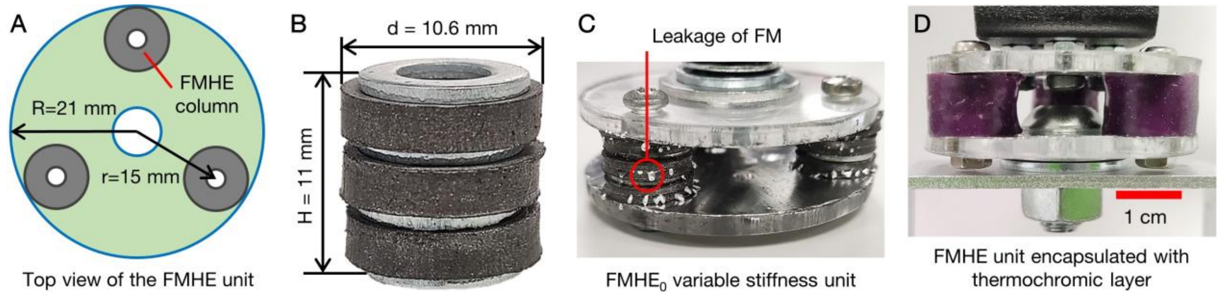

Fig. S19.

Robotic manipulator equipped with the FMHE variable stiffness compensation unit. (**A**) Top view of the FMHE variable stiffness unit, showing the positions of the three FMHE columns. (**B**) Optical image of an FMHE column. (**C**) FM leakage of the $FMHE_0$ variable stiffness unit after being compressed by 60% at 80 °C. (**D**) The FMHE variable stiffness unit encapsulated with a thermochromic Ecoflex layer.

Figure S19, A and B shows the distribution and size of the $FMHE_3$ columns. Three $FMHE_3$ columns (*Φ*10.6×11 mm) are arranged in an equilateral triangle and sandwiched between two electrode plates which are used to apply voltages. Each FMHE column consists of three pieces of $FMHE_3$ samples with two alternately stacked gaskets. The structure of the robotic manipulator equipped with the FMHE variable stiffness compensation unit is shown in Fig. 4B. From top to bottom are the manipulator, the FMHE variable stiffness unit and a digital servo motor with a blocked-rotor torque of 3 N·m.

According to Fig. 3E, $FMHE_0$ has a more significant variable stiffness effect than $FMHE_3$. Theoretically, the variable stiffness unit based on $FMHE_0$ can obtain a higher blocked-rotor angle. However, we still choose to use $FMHE_3$ because of the FM leakage of $FMHE_0$ during compression at a high temperature. As we analysed in Fig. 1B, the PDMS layer between adjacent FM particles

in $FMHE_0$ is very thin. At a high temperature, these thin PDMS layers can rupture at a compressive strain of > 55%, causing leakage of melted FM (see Fig. S19C). In contrast, the PDMS layer in $FMHE_3$ is much thicker, so there is almost no FM leakage during deformation. Therefore, we encapsulate a thermochromic Ecoflex layer on the $FMHE_3$ columns in the variable stiffness unit, as shown in Fig. S19D. This encapsulation layer can not only avoid FM leakage but also indicate the working state of the FMHE compensation unit.

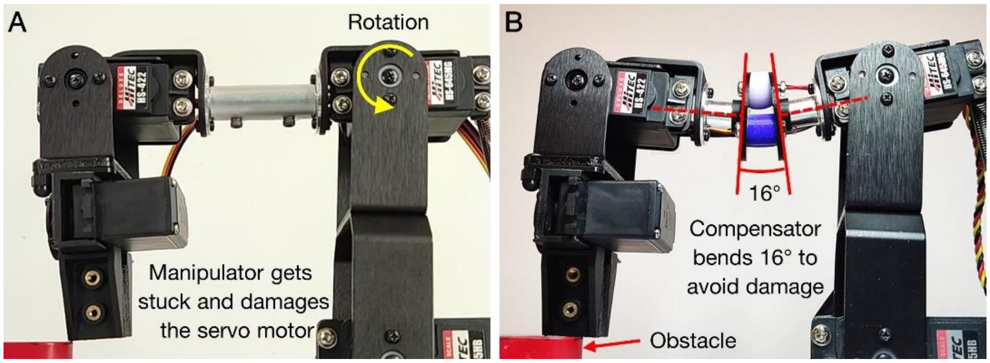

Fig. S20.

Photos of a robotic manipulator installed (A) without and (B) with an FMHE compensator when it is blocked by an obstacle (the red metal block in the photo) during operation.

To demonstrate the application of the FMHE compensator, we install it on a small manipulator with a gripper. When the manipulator encounters an obstacle (the red metal block in the lower left corner of photos) during operation, it will immediately get stuck and even cause damage to the drive servo motor due to the surge of current (fig. S20A and Movies S3 and S4). After installing an FMHE compensator, the compensator will gradually bend to avoid jamming the manipulator (fig. S20B and Movie S3). As its bending angle increases, the compensator will self-trigger to reduce its stiffness. It will then bend further to allow the manipulator to go around the obstacle (Movie S4).

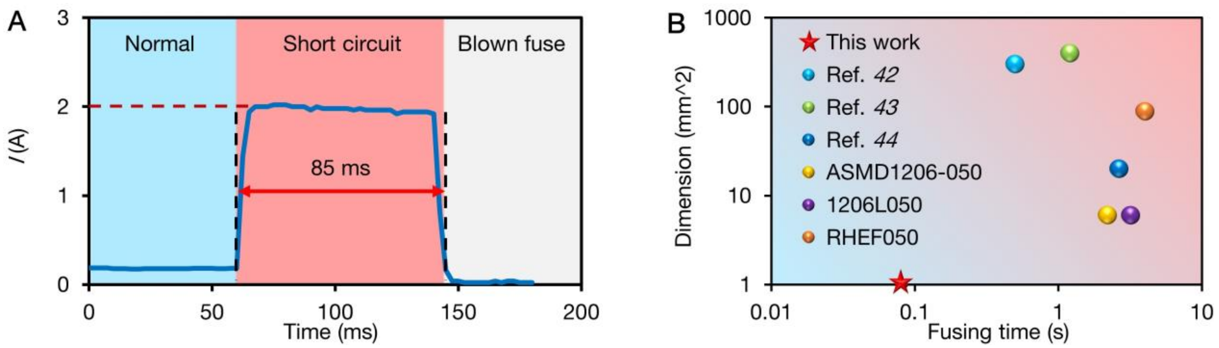

Fig. S21.

A smaller FMHE$_3$ resettable current-limiting fuse. (**A**) Current-time curve of the small FMHE fuse at blowing. The blue, red and grey areas correspond to the normal, short circuit, and blown fuse states, respectively. (**B**) Response time and the dimension of the small FMHE fuse compared to other reported (*44-46*) and commercial (JDTFUSE™ ASMD1206-050, Littelfuse™ 1206L050, Littelfuse™ RHEF050) resettable fuses. The dimension on the y-axis refers to the area of these chip fuses. The short-circuit current during the test is twice the fusing current of each fuse.

By reducing the size of the FMHE resettable fuse, its heating rate during short circuit can be significantly improved due to its higher heating power per unit volume, resulting in a faster response. To demonstrate this, we create a smaller FMHE fuse with FMHE block size of 1×1×1 mm. The FMHE block is pre-compressed by 30% to obtain an initial resistance of ~0.4 Ω and a high fusing resistance of >800 Ω. Fig. S21A shows its current-time curve at blowing. The FMHE fuse is connected in series in a circuit with a working voltage of 6 V and a working current of 0.18 A. At 60 ms, the circuit is manually short-circuited with a fault current of 2 A. With a volumetric heat capacity of 2.06 $J \cdot cm^{-3} \cdot K^{-1}$ and a heating power of 1.6 W, the FMHE fuse will be heated rapidly at a theoretical rate of 0.75 $°C \cdot ms^{-1}$. At 145 ms, the resistance of the FMHE fuse increases

by 2000 times and the current is limited to <10 mA, showing a response time of only 85 ms. Such a fast response speed outperforms state-of-the-art commercial and laboratory prototype PPTC resettable fuses. According to Fig. S21B, the response time of commercial chip resettable fuses with the same working voltage (6 V) and fusing current (1 A) as FMHE fuses is above 1 s. Some reported thermoresponsive fuses can achieve fast response <1 s (*44*, *45*), but their size is much larger than that of FMHE fuses. In conclusion, the FMHE resettable fuse in this work exhibits >10 times faster response and >10 times smaller dimension than state-of-the-art products.

Movie S1.

Deformation of FM particles upon melting in a compressed (20%) $FMHE_0$ sample.

Movie S2.

Deformation of FM particles upon melting in a stretched (12%) $FMHE_0$ sample.

Movie S3.

Manipulators equipped without/with an FMHE compliance compensator encounter an obstacle during moving down.

Movie S4.

Manipulators equipped without/with an FMHE compliance compensator encounter an obstacle during angular movement.

REFERENCES

1. J. A. Faber, A. F. Arrieta, A. R. Studart, Bioinspired spring origami. *Science* **359**, 1386–1391 (2018).

2. D. Rus, M. T. Tolley, Design, fabrication and control of soft robots. *Nature* **521**, 467–475 (2015).

3. Q. Zhong, J. Zhu, F. E. Fish, S. J. Kerr, A. M. Downs, H. Bart-Smith, D. B. Quinn, Tunable stiffness enables fast and efficient swimming in fish-like robots. *Sci. Rob.* **6**, eabe4088 (2021).

4. A. Firouzeh, J. Paik, Grasp mode and compliance control of an underactuated origami gripper using adjustable stiffness joints. *IEEE/ASME Trans. Mechatron.* **22**, 2165–2173 (2017).

5. M. Lanza, A. Sebastian, W. D. Lu, M. le Gallo, M.-F. Chang, D. Akinwande, F. M. Puglisi, H. N. Alshareef, M. Liu, J. B. Roldan, Memristive technologies for data storage, computation, encryption, and radio-frequency communication. *Science* **376**, eabj9979 (2022).

6. J. Lv, Gurunathan Thangavel, Y. Li, J. Xiong, D. Gao, J. Ciou, Matthew Wei Ming Tan, I. Aziz, S. Chen, J. Chen, X. Zhou, W. C. Poh, P. S. Lee, Printable elastomeric electrodes with sweat-enhanced conductivity for wearables. *Sci. Adv.* **7**, eabg8433 (2021).

7. N. Ogihara, N. Ohba, Y. Kishida, On/off switchable electronic conduction in intercalated metal-organic frameworks. *Sci. Adv.* **3**, e1603103 (2017).

8. X. Yu, T. J. Marks, A. Facchetti, Metal oxides for optoelectronic applications. *Nat. Mater.* **15**, 383–396 (2016).

9. E. J. Yoo, M. Lyu, J.-H. Yun, C. J. Kang, Y. J. Choi, L. Wang, Resistive switching behavior in organic–inorganic hybrid $CH_3NH_3PbI_{3-x}Cl_x$ perovskite for resistive random access memory devices. *Adv. Mater.* **27**, 6170–6175 (2015).

10. Z. Yao, L. Pan, L. Liu, J. Zhang, Q. Lin, Y. Ye, Z. Zhang, S. Xiang, B. Chen, Simultaneous implementation of resistive switching and rectifying effects in a metal-organic framework with switched hydrogen bond pathway. *Sci. Adv.* **5**, eaaw4515 (2019).

11. A. A. Talin, A. Centrone, A. C. Ford, M. E. Foster, V. Stavila, P. Haney, R. A. Kinney, V. Szalai, F. el Gabaly, H. P. Yoon, F. Léonard, M. D. Allendorf, Tunable electrical conductivity in metal-organic framework thin-film devices. *Science* **343**, 66–69 (2014).

12. Y. Wei, Y. Chen, T. Ren, Q. Chen, C. Yan, Y. Yang, Y. Li, A novel, variable stiffness robotic gripper based on integrated soft actuating and particle jamming. *Soft Rob.* **3**, 134–143 (2016).

13. S. Jadhav, M. R. A. Majit, B. Shih, J. P. Schulze, M. T. Tolley, Variable stiffness devices using fiber jamming for application in soft robotics and wearable haptics. *Soft Rob.* **9**, 173–186 (2022).

14. J. Yang, S. S. Sun, H. Du, W. H. Li, G. Alici, H. X. Deng, A novel magnetorheological elastomer isolator with negative changing stiffness for vibration reduction. *Smart Mater. Struct.* **23**, 105023 (2014).

15. S. Sun, H. Deng, H. du, W. Li, J. Yang, G. Liu, G. Alici, T. Yan, A Compact variable stiffness and damping shock absorber for vehicle suspension. *IEEE/ASME Trans. Mechatron.* **20**, 2621–2629 (2015).

16. A. Loeve, P. Breedveld, J. Dankelman, Scopes too flexible...and too stiff. *IEEE Pulse* **1**, 26–41 (2010).

17. T. Ranzani, G. Gerboni, M. Cianchetti, A. Menciassi, A bioinspired soft manipulator for minimally invasive surgery. *Bioinspir. Biomim.* **10**, 035008 (2015).

18. J. A. Rogers, T. Someya, Y. Huang, Materials and mechanics for stretchable electronics. *Science* **327**, 1603–1607 (2010).

19. B. Wang, A. Facchetti, Mechanically flexible conductors for stretchable and wearable E-skin and E-textile devices. *Adv. Mater.* **31**, 1901408 (2019).

20. H. Joo, D. Jung, S.-H. Sunwoo, J. H. Koo, D.-H. Kim, Material design and fabrication strategies for stretchable metallic nanocomposites. *Small* **16**, 1906270 (2020).

21. H. Liu, Y. Xin, H. K. Bisoyi, Y. Peng, J. Zhang, Q. Li, Stimuli-driven insulator–conductor transition in a flexible polymer composite enabled by biphasic liquid metal. *Adv. Mater.* **33**, 2104634 (2021).

22. S. Chen, H.-Z. Wang, X.-Y. Sun, Q. Wang, X.-J. Wang, L.-B. Chen, L.-J. Zhang, R. Guo, J. Liu, Generalized way to make temperature tunable conductor–insulator transition liquid metal composites in a diverse range. *Mater. Horiz.* **6**, 1854–1861 (2019).

23. B. E. Schubert, D. Floreano, Variable stiffness material based on rigid low-melting-point-alloy microstructures embedded in soft poly(dimethylsiloxane) (PDMS). *RSC Adv.* **3**, 24671–24679 (2013).

24. Y. Lin, J. Genzer, M. D. Dickey, Attributes, fabrication, and applications of gallium-based liquid metal particles. *Adv. Sci.* **7**, 2000192 (2020).

25. Y. Piskarev, J. Shintake, V. Ramachandran, N. Baugh, M. D. Dickey, D. Floreano, Lighter and stronger: Cofabricated electrodes and variable stiffness elements in dielectric actuators. *Adv. Intell. Syst.* **2**, 2000069 (2020).

26. A. Tonazzini, S. Mintchev, B. Schubert, B. Mazzolai, J. Shintake, D. Floreano, Variable stiffness fiber with self-healing capability. *Adv. Mater.* **28**, 10142–10148 (2016).

27. Y. Hao, J. Gao, Y. Lv, J. Liu, Low melting point alloys enabled stiffness tunable advanced materials. *Adv. Funct. Mater.* **32**, 2201942 (2022).

28. L. J. Briggs, Gallium: Thermal conductivity; supercooling; negative pressure. *J. Chem. Phys.* **26**, 784–786 (1957).

29. S.-Y. Tang, D. R. G. Mitchell, Q. Zhao, D. Yuan, G. Yun, Y. Zhang, R. Qiao, Y. Lin, M. D. Dickey, W. Li, Phase separation in liquid metal nanoparticles. *Matter* **1**, 192–204 (2019).

30. S. Chen, R. Zhao, X. Sun, H. Wang, L. Li, J. Liu, Toxicity and biocompatibility of liquid metals. *Adv. Healthc. Mater.* 2201924 (2022).

31. D. Lee, H. Lee, Y. Jeong, Y. Ahn, G. Nam, Y. Lee, Highly sensitive, transparent, and durable pressure sensors based on sea-urchin shaped metal nanoparticles. *Adv. Mater.* **28**, 9364–9369 (2016).

32. S. Stassi, G. Canavese, Spiky nanostructured metal particles as filler of polymeric composites showing tunable electrical conductivity. *J Polym Sci B* **50**, 984–992 (2012).

33. A. Zavabeti, J. Z. Ou, B. J. Carey, N. Syed, R. Orrell-Trigg, E. L. H. Mayes, C. Xu, O. Kavehei, A. P. O'Mullane, R. B. Kaner, K. Kalantar-zadeh, T. Daeneke, A liquid metal reaction environment for the room-temperature synthesis of atomically thin metal oxides. *Science* **358**, 332–335 (2017).

34. P. Anton, K. Uwe, M. Hartmut, P. Johann, *In situ* x-ray reflectivity study of the oxidation kinetics of liquid gallium and the liquid alloy. *J. Phys. Condens. Matter* **10**, 971–982 (1998)..

35. G. Yun, S.-Y. Tang, Q. Zhao, Y. Zhang, H. Lu, D. Yuan, S. Sun, L. Deng, M. D. Dickey, W. Li, Liquid metal composites with anisotropic and unconventional piezoconductivity. *Matter* **3**, 824–841 (2020).

36. G. Yun, S.-Y. Tang, H. Lu, S. Zhang, M. D. Dickey, W. Li, Hybrid-filler stretchable conductive composites: From fabrication to application. *Small Sci.* **1**, 2000080 (2021).

37. A. Müller, M. C. Wapler, U. Wallrabe, A quick and accurate method to determine the Poisson's ratio and the coefficient of thermal expansion of PDMS. *Soft Matter* **15**, 779–784 (2019).

38. S. Çınar, I. D. Tevis, J. Chen, M. Thuo, Mechanical fracturing of core-shell undercooled metal particles for heat-free soldering. *Sci. Rep.* **6**, 21864 (2016).

39. S. Cantournet, R. Desmorat, J. Besson, Mullins effect and cyclic stress softening of filled elastomers by internal sliding and friction thermodynamics model. *Int. J. Solids Struct.* **46**, 2255–2264 (2009).

40. Y. Zhang, H. Lu, D. T. Pham, Y. Wang, M. Qu, J. Lim, S. Su, Peg–hole disassembly using active compliance. *R. Soc. Open Sci.* **6**, 190476 (2019).

41. M. A. Lee, Y. Zhu, P. Zachares, M. Tan, K. Srinivasan, Making sense of vision and touch: Learning multimodal representations for contact-rich tasks. *IEEE Trans. Rob.* **36**, 582–596 (2020).

42. S. Ohtsuka, H. Suetomi, M. Hikita, Fundamental properties of OFF-ON resistance of a new type self-recovering fuse operated by dielectrophoresis, in *Proceedings of the Conference Record of the 2006 Twenty-Seventh International Power Modulator Symposium* (2006), pp. 474–477.

43. C. Niu, B. Wang, H. He, Y. Wu, M. Rong, J. Li, L. Wang, P. Zhao, A novel liquid metal fault current limiter based on active trigger method. *IEEE Trans. Power Deliv.* **36**, 3619–3628 (2021).

44. Z. Chen, P-C. Hsu, J. Lopez, Y. Li, J. W. F. To, N. Liu, C. Wang, S. C. Andrews, J. Liu, Y. Cui, Z. Bao, Fast and reversible thermoresponsive polymer switching materials for safer batteries. *Nat. Energy* **1**, 15009 (2016).

45. S. Wang, S. Ma, L. Cao, Q. Li, Q. Ji, J. Huang, N. Lu, X. Xu, Y. Liu, J. Zhu, Conductive vitrimer nanocomposites enable advanced and recyclable thermo-sensitive materials. *J. Mater. Chem. C* **8**, 11681–11686 (2020).

46. R. Yang, Y. Wang, D. Wu, Y. Deng, Y. Luo, X. Cui, X. Wang, Z. Shu, C. Yang, Low-temperature fusible silver micro/nanodendrites-based electrically conductive composites for next-generation printed fuse-links. *ACS Nano* **11**, 7710–7718 (2017).
